# A Closed-Loop Quality Improvement Intervention to Enhance Diabetic Foot Risk Assessment Compliance at Atbara Teaching Hospital

**DOI:** 10.7759/cureus.110000

**Published:** 2026-05-31

**Authors:** Waad Abdalazim Abdalwadod Gadora, Esra Abdalla, Mohammedomer Eltayeb, Saad Adam Abdallah Abakar, Hamdi Bashar Ahmed Ibrahim, Mustafa Sabir Abakar Awad, Amna Maawia Badawi Abdalla, Mohamed Elmuntasir Salah, Fatima Abdelwahab Ali Ahmed, Nemah Abdulhameed Ali Saleh Dejrah, Malath Salih Younis Bashiri, Mohamed Haj Hassan, Rmmah Ali Yahyea, Ahmed Kamal Ahmed Hasanin, Mohamed Osman Mohamed Idres, Abdelrahman Sahnon, Wadah Mohamed Hussein Mohamed

**Affiliations:** 1 Emergency Medicine, University of Gezira, Wad Madani, SDN; 2 Medicine and Surgery, Bashair University Hospital, Khartoum, SDN; 3 Emergency Medicine, Atbara Teaching Hospital, Atbara, SDN; 4 Medicine, Al Neelain University, Khartoum, SDN; 5 Internal Medicine, Sudan Medical Specialization Board, Khartoum, SDN; 6 Medicine, College of Medicine, Sudan University of Science and Technology, Khartoum, SDN; 7 Emergency Medicine, Port Sudan Teaching Hospital, Port Sudan, SDN; 8 Medicine and Surgery, Sudan International University, Khartoum, SDN; 9 Surgery, Royal College of Surgeons, London, GBR; 10 Internal Medicine, Sligo University Hospital, Sligo, IRL

**Keywords:** clinical audit, diabetic foot, hospital documentation, neuropathy screening, peripheral arterial disease, quality improvement, risk assessment

## Abstract

Background and objective

Diabetic foot complications are a major cause of morbidity and preventable lower limb amputation among patients with diabetes mellitus. Despite clear international guidelines recommending routine structured foot risk assessment, adherence in hospital settings remains inconsistent. The objective of this study was to evaluate baseline compliance with diabetic foot risk assessment documentation and determine the impact of implementing a structured assessment tool and targeted staff education.

Methods

A closed-loop quality improvement project was conducted at Atbara Teaching Hospital. The first audit cycle (August 2025) included 50 hospitalized adult patients with diabetes mellitus. Following baseline assessment, a standardized diabetic foot risk assessment checklist was introduced with staff orientation and workflow integration over a two-month intervention period. A second prospective audit cycle (November to December 2025) assessed 50 consecutive patients using identical criteria. Compliance with guideline-recommended assessment components was compared between cycles using chi-square testing.

Results

Baseline documentation of key risk assessment components was low, particularly for neuropathy (8; 16%), peripheral arterial disease (3; 6%), and risk stratification (0; 0%). Following intervention, compliance improved significantly across all clinical domains, reaching 50 (100%) for neuropathy assessment, vascular assessment, risk score calculation, and follow-up planning (p < 0.001). Documentation of patient identifiers showed minimal change, except for file number recording, which declined significantly from 45 (90%) to 28 (56%) (p < 0.001).

Conclusions

Implementation of a structured assessment tool combined with targeted education significantly improved compliance with diabetic foot risk assessment standards. Structured quality improvement strategies represent an effective approach to bridging gaps between guideline recommendations and routine clinical practice.

## Introduction

Diabetes mellitus is a major global public health challenge, affecting hundreds of millions of adults worldwide and contributing to a range of microvascular and macrovascular complications. One of the most devastating complications is the development of diabetic foot ulcers (DFUs), which are chronic wounds that occur as a result of peripheral neuropathy, peripheral arterial disease, or a combination of structural foot abnormalities and sustained pressure. DFUs are associated with substantial morbidity and mortality, and they significantly increase healthcare utilization and costs due to prolonged treatment and frequent hospital admissions. The lifetime incidence of DFUs in people with diabetes is estimated at 19-34%, and affected individuals face a markedly increased risk of lower limb amputation, disability, and premature death compared with those without foot complications [1,2].

The pathophysiological mechanisms underlying diabetic foot disease are multifactorial. Peripheral sensory neuropathy diminishes protective sensation in the feet, while peripheral arterial disease compromises blood flow, impairing tissue oxygenation and wound healing. These processes predispose the diabetic foot to ulceration even in the absence of significant trauma. Secondary complications such as infection, gangrene, sepsis, and critical limb ischemia are common and contribute to both limb loss and increased mortality. Peripheral neuropathy affects approximately half of adults with diabetes during their lifetime, and PAD is highly prevalent among those with foot complications [3,4].

The global burden of diabetic foot complications extends beyond individual morbidity; it imposes a heavy economic strain on health systems. In high-income countries, diabetic foot problems account for a disproportionate share of direct diabetes care costs, with expenditures rivaling those for major chronic diseases. Additionally, delayed healing and recurrent ulceration increase the lifetime healthcare costs for affected individuals. These observations have underscored the importance of preventive strategies and early intervention to minimize adverse outcomes, reduce hospital stays, and limit the need for costly surgical procedures [1,5].

Evidence-based international clinical practice guidelines, such as those developed by the International Working Group on the Diabetic Foot (IWGDF), emphasize the importance of routine foot risk assessment as a cornerstone of diabetic care. These guidelines recommend periodic comprehensive foot examinations to identify loss of protective sensation, signs of peripheral arterial disease, foot deformities, and history of prior ulceration or amputation. By stratifying patients according to risk level, clinicians can tailor the frequency of follow-up and implement targeted preventive interventions aimed at reducing ulcer occurrence and progression [1,6].

Despite clear guideline recommendations and the known benefits of early risk detection, structured foot assessment and risk stratification are inconsistently implemented in clinical practice, particularly in resource-limited settings, including Sudan and the broader sub-Saharan African region. In Sudan, the prevalence of diabetic foot ulceration has been reported at 18.1%, significantly higher than many global and regional estimates, reflecting advanced disease at presentation and gaps in preventive care structures. These data highlight a critical gap in early foot assessment practices and documentation in routine clinical care that, if addressed, could mitigate morbidity, reduce amputations, and improve health outcomes. Accordingly, the general objective of this clinical audit was to evaluate current practice in diabetic foot risk assessment among hospitalized adult patients with diabetes mellitus against international guideline standards, with a focus on completeness of documentation and identification of key risk factors. The specific objectives were to quantify baseline compliance with routine foot assessments, implement a structured clinical assessment tool with staff education, and measure changes in audit indicators in a reaudit cycle after intervention implementation [7].

## Materials and methods

This project was designed as a closed-loop quality improvement initiative conducted at Atbara Teaching Hospital, a secondary-level public referral hospital in River Nile State, Sudan. The project aimed to evaluate and enhance compliance with standardized diabetic foot risk assessment among hospitalized adult patients with diabetes mellitus. The audit followed a two-cycle framework consisting of baseline evaluation, implementation of structured interventions, and reassessment to determine the impact of the quality improvement measures.

The study was carried out in the adult medical wards where patients with diabetes are routinely admitted for acute and chronic medical conditions. All adult patients aged 18 years and above with a documented diagnosis of type 1 or type 2 diabetes mellitus admitted during the audit periods were eligible for inclusion. Patients admitted for less than 24 hours, or those with preexisting major lower limb amputation, were excluded to ensure consistent evaluation of foot risk assessment practices.

The audit standard was derived from the 2023 IWGDF guidelines, which recommend routine structured foot risk assessment for all individuals with diabetes. The standard required documentation of neuropathy assessment, evaluation for peripheral arterial disease, inspection for foot deformities and skin integrity, documentation of previous ulceration or amputation, infection screening, footwear assessment, evaluation of patient self-care capacity, calculation of overall risk score, classification of risk level, and documentation of an appropriate follow-up plan [6]. In alignment with international recommendations, the target compliance rate for each assessment component was set at 100%.

The first audit cycle was conducted over a one-month period from August 1 to August 31, 2025. During this phase, a retrospective review of consecutive medical records of eligible diabetic patients admitted during the study period was performed. Documentation practices were assessed against the predefined audit criteria to establish baseline compliance. A total coverage sampling approach was used, whereby all eligible diabetic patients admitted during the audit period were included. This resulted in 50 patients being included in the first audit cycle.

Following analysis of the baseline findings, an intervention phase was initiated beginning on September 1, 2025 and continued for two months until October 31, 2025. During this period, a structured diabetic foot risk assessment checklist was developed and formally introduced into routine clinical practice. The intervention included targeted staff education sessions, orientation meetings to reinforce guideline-based assessment, distribution of standardized scoring sheets, placement of visual reminders within clinical areas, and structured feedback to clinical teams regarding identified gaps in practice. The checklist was integrated into patient admission documentation to facilitate routine use and ensure sustainability.

The second audit cycle was conducted prospectively over a two-month period from November 1, 2025 to December 31, 2025. Consecutive eligible patients admitted during this period were evaluated using the same inclusion criteria and assessment standards to determine post-intervention compliance with the standardized diabetic foot risk assessment components. Data collection in both cycles was performed using a structured data extraction form aligned with the assessment checklist to ensure methodological consistency. Data were extracted independently by two reviewers, and disagreements were resolved through consensus. Inter-rater reliability was not formally assessed. Similarly, all eligible patients admitted during the reaudit period were included, resulting in 50 patients in the second audit cycle.

The primary outcome measure was the proportion of patient records demonstrating complete documentation of the recommended diabetic foot risk assessment components. Secondary outcomes included compliance with individual clinical assessment parameters, documentation of risk stratification, and recording of a follow-up plan. Data were entered into Microsoft Excel (Microsoft Corporation, Redmond, WA, USA) and analyzed using descriptive statistics. Categorical variables were expressed as frequencies and percentages, and improvement between cycles was assessed by comparing compliance rates before and after implementation of the intervention.

The project was conducted in accordance with institutional governance policies for quality improvement initiatives at Atbara Teaching Hospital, Sudan, and was approved by the hospital’s clinical governance and quality committee under reference number ATB/IRB/2025/018. No alterations were made to standard patient care. All data were anonymized prior to analysis to ensure patient confidentiality, and access to clinical records was restricted to authorized members of the project team. As the initiative was classified as a service evaluation and quality improvement project, formal ethical committee review was not required under local regulatory frameworks. The study was conducted in accordance with the principles of the Declaration of Helsinki. Although exempt from formal ethical review as a quality improvement project, the principles of the Declaration of Helsinki regarding patient confidentiality and data protection were respected throughout the study.

## Results

A total of 100 patient records were reviewed across two audit cycles, with 50 patients included in each cycle. Baseline documentation of general patient identifiers was relatively high for patient name, age, and date of documentation. Following implementation of the structured assessment tool, documentation of patient name, age, and date reached full compliance at 50 (100%), although these changes did not achieve statistical significance. Documentation of file number, however, declined significantly from 45 (90%) in the first cycle to 28 (56%) in the second cycle (χ² = 14.66, df = 1, p < 0.001). Recording of clinician name and department showed slight reductions between cycles but remained statistically nonsignificant.

In contrast, documentation of core clinical risk assessment components demonstrated substantial and statistically significant improvement following implementation of the intervention. Table 1 demonstrates marked improvement across nearly all assessment domains after the introduction of the standardized diabetic foot risk assessment checklist. The most notable improvements were observed in risk stratification parameters, including calculation of total risk score and classification of risk level, both of which improved from 0 (0%) at baseline to 50 (100%) after intervention (p < 0.001). Similarly, documentation of foot deformity and skin assessment increased significantly from 1 (2%) in the first cycle to 50 (100%) in the second cycle (p < 0.001). Significant improvements were also observed in neuropathy assessment, peripheral arterial disease evaluation, ulcer and amputation history documentation, infection screening, footwear assessment, patient self-care capacity evaluation, and structured follow-up planning.

**Table 1:** Compliance with diabetic foot risk assessment components before and after intervention (n = 50 per cycle) This table presents the frequency and percentage of documented diabetic foot risk assessment components during the baseline audit cycle (August 1-31, 2025) and the reaudit cycle (November 1 to December 31, 2025) following implementation of the quality improvement intervention. Comparisons between audit cycles were performed using the Pearson chi-square test. A p-value of < 0.05 was considered statistically significant. For comparisons involving zero events in the first audit cycle, chi-square approximations may be unstable; Fisher’s exact test yielded equivalent p-values < 0.001.

Assessment component	Cycle 1, n (%)	Cycle 2, n (%)	χ²	p-value
Patient identifiers				
Patient name documented	47 (94%)	50 (100%)	3.09	0.078
Age documented	47 (94%)	50 (100%)	3.09	0.078
File number documented	45 (90%)	28 (56%)	14.66	<0.001
Date documented	49 (98%)	50 (100%)	1.01	0.315
Clinician and department documented	47 (94%)	43 (86%)	1.78	0.182
Clinical risk assessment				
Neuropathy assessment	8 (16%)	50 (100%)	72.41	<0.001
Peripheral arterial disease assessment	3 (6%)	50 (100%)	88.7	<0.001
Foot deformity/skin inspection	1 (2%)	50 (100%)	96.07	<0.001
Ulcer/amputation history	1 (2%)	50 (100%)	96.07	<0.001
Infection/skin integrity assessment	0 (0%)	50 (100%)	100	<0.001
Footwear assessment	0 (0%)	50 (100%)	100	<0.001
Follow-up access factors	0 (0%)	50 (100%)	100	<0.001
Patient self-care capacity	0 (0%)	47 (94%)	88.7	<0.001
Risk stratification and planning				
Total risk score calculated	0 (0%)	50 (100%)	100	<0.001
Risk level classified	0 (0%)	50 (100%)	100	<0.001
Follow-up plan documented	11 (22%)	50 (100%)	63.93	<0.001

Overall, the introduction of a standardized diabetic foot risk assessment tool combined with structured staff orientation was associated with a dramatic and statistically significant improvement in documentation of guideline-recommended assessment components, risk stratification, and follow-up planning (Table 1).

## Discussion

This clinical audit demonstrates that implementation of a structured diabetic foot assessment tool combined with targeted staff education dramatically improved documentation compliance for key risk factors in hospitalized adult patients with diabetes. The baseline cycle revealed substantial deficiencies in essential components of diabetic foot assessment, including neuropathy screening, vascular evaluation, and risk stratification, which were either poorly documented or entirely absent. These findings identify significant gaps in practice, consistent with previous reports showing that routine diabetic foot assessment remains under-implemented in many clinical settings despite clear guideline recommendations. For example, audits in both primary and secondary care contexts have uncovered widespread variability in foot examination practices and documentation, indicating systemic deficiencies that require structured interventions [8].

International clinical practice guidelines strongly advocate routine and systematic assessment of the diabetic foot to identify individuals at risk of ulcers and other complications. The IWGDF guidelines provide comprehensive recommendations for assessing sensory neuropathy, peripheral arterial disease, foot deformities, and other pertinent risk factors, with an emphasis on structured documentation in clinical practice [6]. Similarly, the NICE diabetic foot guideline underscores the need for regular foot examinations for people with diabetes to reduce variation in practice and to guide preventive care. Despite these well-established standards, real-world practice often falls short, particularly in resource-limited environments where time constraints, competing priorities, and lack of standardized tools can impede guideline adherence [9].

Successful interventions to improve diabetic foot assessment have been described in quality improvement literature, with structured approaches yielding significant gains in documentation and clinical practice. A quality improvement project that introduced standardized screening procedures demonstrated increased rates of annual foot examinations and improved early detection of at-risk patients, supporting the utility of systematic tools paired with provider training [10]. Similarly, other implementation studies have shown that embedding diabetic foot screening tools into clinical workflows, including electronic medical records or primary care pathways, can noticeably enhance provider adherence to assessment recommendations and reduce variability in practice [11]. The intervention in the present audit was implemented using locally available resources, including brief staff education sessions, printed assessment checklists, and visual reminders within the medical wards. No additional external funding was required, supporting the feasibility of similar quality improvement interventions in resource-limited healthcare settings.

The findings from this audit also align with recent primary research outside the local context. In Gauteng Province, South Africa, direct clinical assessments revealed inadequate foot screening practices and inconsistent use of risk stratification, further underscoring the gap between guideline recommendations and real-world practice in diverse healthcare environments [12]. These findings are consistent with reports from other low-resource settings within sub-Saharan Africa, where diabetic foot assessment and risk stratification remain inconsistently implemented despite established guideline recommendations. Furthermore, methodological reviews of diabetic foot clinical practice guidelines have highlighted variability in guideline quality and implementation, pointing to a broader need for harmonized, high-quality standards that are feasible to adopt across settings [13]. These insights reinforce the importance of both guideline clarity and implementation support to bridge gaps between recommended care and practice.

Although this audit focused on process measures rather than long-term clinical outcomes, improvement in documentation is a critical step toward enhanced patient care. Structured assessment enables early identification of patients at high risk for foot complications, which can inform individualized management plans, preventive education, and appropriate referrals. Furthermore, improved documentation creates an accurate dataset on which future outcome evaluations, such as ulcer incidence, infection rates, and amputation rates, can be reliably based. Sustainability of these improvements may require periodic reaudits, continued reinforcement of guideline-based practices, and incorporation of diabetic foot assessment training into staff orientation programs. Future research should investigate whether sustained improvements in assessment translate into measurable reductions in adverse diabetic foot outcomes, especially in settings with high disease burdens and constrained healthcare resources [14,15].

This study has several limitations that should be acknowledged. First, as a single-center clinical audit conducted within one hospital setting, the generalizability of the findings to other institutions or healthcare systems may be limited. Second, the audit primarily assessed documentation practices rather than direct clinical performance; therefore, improvements in recorded data may not fully reflect actual changes in clinical behavior or patient care delivery. Third, the post-intervention assessment was conducted over a relatively short period, and thus the sustainability of the observed improvements over time remains uncertain. Additionally, staff awareness of the reaudit cycle may have inflated post-intervention compliance rates (Hawthorne effect), and the observed improvements may therefore be lower under routine unobserved clinical conditions [16]. Finally, no assessment of inter-rater reliability was performed, which may introduce variability in how documentation was interpreted during data collection.

In summary, this quality improvement project demonstrates that a structured risk assessment tool, combined with targeted education and integration into clinical practice, can substantially improve documentation of diabetic foot risk factors. These results emphasize the need for standardized assessment frameworks and support systems to ensure adherence to guideline recommendations and provide a model that can be adapted to similar clinical environments facing challenges in diabetic foot care.

## Conclusions

Implementation of a structured diabetic foot risk assessment tool significantly improved compliance with guideline-recommended documentation in hospitalized patients with diabetes. Baseline assessment practices were inconsistent and incomplete, particularly in key domains such as neuropathy evaluation, vascular assessment, and risk stratification. Following targeted education and integration of a standardized checklist into routine practice, documentation rates improved markedly across all clinical components.

These findings demonstrate that structured quality improvement interventions can effectively bridge the gap between recommended standards and real-world practice. Sustained monitoring and periodic reaudits are essential to maintain improvements and support long-term enhancement of diabetic foot care. Integration of standardized diabetic foot risk assessment checklists into routine inpatient documentation, regular staff education and orientation on guideline-based assessment practices, and continued periodic reaudits may further support long-term sustainability and improvement in diabetic foot care delivery.
